# A Review of Research on the Valorization and Risk Management of Municipal Solid Waste Incineration Bottom Ash

**DOI:** 10.3390/ma19071471

**Published:** 2026-04-07

**Authors:** Yang Nan, Wenli Wang, Haozhe Chen, Jiapeng Guo, Yanqiang Chen, Du Yuan

**Affiliations:** State Key Laboratory of Eco-Hydraulics in Northwest Arid Region, Xi’an University of Technology, Xi’an 710048, China; 2240720027@stu.xaut.edu.cn (Y.N.); 105648@xaut.edu.cn (H.C.); 2240721103@stu.xaut.edu.cn (J.G.); 2240720044@stu.xaut.edu.cn (Y.C.); 2240720075@stu.xaut.edu.cn (D.Y.)

**Keywords:** MSWIBA, high-value, risk management, heavy metal properties, synergistic pathways

## Abstract

**Highlights:**

**What are the main findings?**

- MSWIBA is highly heterogeneous; its physicochemical properties and heavy metal speciation are significantly influenced by waste source, particle size, and seasonal variation, with heavy metals (Pb, Zn, Cu) enriched in fine particles (<0.5 mm).
- MSWIBA can be used as a construction material, but high substitution rates reduce mechanical strength due to its porous structure and reactive aluminum; Fe, Al, Cu, and rare earths can be recovered via physical separation and hydrometallurgy, though efficiency for fine particles involves a trade-off with cost.
- Solidification/stabilization (cement, alkali activation, thermal treatment) and pretreatment (washing, acid leaching) effectively reduce heavy metal leaching, but single technologies struggle to balance resource recovery with environmental safety, and long-term stability data remain insufficient.

**What are the implications of the main findings?**

- Targeted segregation of MSWIBA based on particle size and composition is necessary to enable context-specific valorization strategies.
- A synergistic “recovery-stabilization-utilization” integration framework should be established to resolve the inherent conflict between resource recovery and detoxification through technological coupling.
- Priority should be given to developing intelligent sorting, elucidating long-term immobilization mechanisms, and conducting life cycle assessments to support the transition of MSWIBA into a sustainable resource.

**Abstract:**

Municipal solid waste incineration bottom ash (MSWIBA) represents both a resource opportunity and an environmental challenge in waste-to-energy systems. This comprehensive review examines MSWIBA’s physicochemical properties, heavy metal behavior, and applications in construction materials, alongside metal recovery techniques and risk mitigation strategies. The research introduces an integrated management framework combining property assessment with coordinated stream processing to reconcile resource recovery with environmental safety. Future studies should focus on advanced analytical methods, hybrid processes, long-term immobilization mechanisms, and life cycle assessment. These innovations aim to transform MSWIBA into a sustainable resource, supporting circular economy principles and low-carbon development.

## 1. Introduction

As global urbanization intensifies, the escalating volume of municipal solid waste (MSW) presents a formidable threat to both environmental systems and public health. Waste-to-energy (WtE) incineration has emerged as a leading MSW management strategy worldwide, particularly in nations where land scarcity and high energy demand are critical issues. This technology effectively reduces waste volume and recovers energy. Nevertheless, the process does not eliminate waste but rather transforms it into residues, with Municipal solid waste incineration bottom ash (MSWIBA) being the most significant byproduct. Comprising 15–25% of the original waste mass, MSWIBA is produced in vast and ever-increasing quantities [1,2]. This material is chemically and physically complex, containing a mixture of glass, ceramics, metals, unburned carbon, and mineral oxides. While MSWIBA is often considered relatively stable, it is known to concentrate heavy metals, including copper (Cu), zinc (Zn), lead (Pb), cadmium (Cd), chromium (Cr), and nickel (Ni). The potential for these metals to leach into the environment under certain conditions creates a significant risk for soil and groundwater contamination [2,3,4]. Consequently, developing methods for the high-value utilization of MSWIBA while ensuring environmental safety has become a central and challenging focus in solid waste valorization research.

The principal avenue for the high-value utilization of MSWIBA is in the construction sector, where it serves as a supplementary raw material in the production of cement, concrete, mortar, road bases, and bricks [5,6,7,8,9]. Furthermore, MSWIBA contains valuable metals like aluminum (Al), copper (Cu), iron (Fe), and zinc (Zn), as well as trace amounts of rare earth elements (REEs) and precious metals (e.g., gold, silver), offering significant potential for resource recovery and associated economic and environmental benefits [1,10,11,12,13]. The widespread adoption of these applications, however, is contingent upon rigorous environmental risk mitigation, primarily the effective containment of heavy metal leaching. Various techniques, including solidification/stabilization (S/S), physical and chemical pretreatments (such as washing, acid leaching, and alkali fusion), and adsorption, have been developed to reduce the mobility and bioavailability of these hazardous elements [14,15,16].

Despite substantial research progress, the large-scale engineering application of MSWIBA remains hindered by its intrinsic complexity, the lack of standardized technical approaches, and uncertainty regarding its long-term environmental behavior. Current literature tends to focus narrowly on specific utilization pathways or risk control measures, failing to provide a holistic, integrated analysis of the entire value chain, i.e., from material properties to end-use applications to long-term risk assessment. Therefore, this review aims to provide a comprehensive assessment of the current state of MSWIBA valorization and risk control. First, it will analyze the physicochemical characteristics of MSWIBA, with a particular focus on the speciation and key factors influencing the leaching behavior of heavy metals. Second, it will systematically evaluate the technical feasibility, performance, and limitations of its two main application streams: as a construction material and for the recovery of valuable metals. Third, it will critically assess the mechanisms, efficacy, and challenges of various risk control technologies. Finally, by synthesizing the existing knowledge, this review will identify contradictions in current management paradigms and propose a systematic, integrated framework and future research directions aligned with circular economy principles and sustainable management. The objective is to offer a robust theoretical foundation and practical guidance for the safe and efficient resource recovery and management of MSWIBA.

## 2. Characteristics of MSWIBA

The properties of MSWIBA form the basis of its valorization and risk management. A thorough understanding of MSWIBA’s physical, chemical, and mineralogical composition, along with the speciation and leaching behavior of its heavy metals, is essential for the development of effective treatment and valorization technologies. These properties are not static; they are subject to variation due to a multitude of factors, such as the composition of the original waste, the parameters of the incineration process, and the methods used for post-combustion ash treatment.

### 2.1. Physicochemical Composition and Mineralogical Properties

MSWIBA is a highly heterogeneous material whose physical composition typically comprises glass, ceramics, metals (ferrous and non-ferrous), unburned organic matter, mineral particles, and a minor amount of slag. This inherent heterogeneity presents the primary challenge for MSWIBA utilization. Chemically, MSWIBA is predominantly composed of oxygen and calcium (Table 1), with its main constituents being silicon dioxide (SiO_2_), calcium oxide (CaO), aluminum oxide (Al_2_O_3_), and iron oxide (Fe_2_O_3_)—key components in cement and concrete production [17,18]. However, MSWIBA also contains chlorides, sulfates, and alkali metal oxides, which can adversely affect material performance and environmental safety. For instance, chlorides can induce corrosion in reinforcing steel and must be reduced to acceptable levels through pre-treatment methods like alkali fusion and hydrothermal techniques [15].

From a mineralogical perspective, analyses using XRPD and XRF have shown that MSWIBA generally contains crystalline phases such as quartz, calcite, calcium silicate, anorthite, and magnetite, alongside a significant amorphous phase. This mineralogical endowment confers a certain pozzolanic activity and cementitious potential, indicating its potential application in cement-based materials [19]. Bayuseno and Schmahl (2010) [20], through XRPD, polarized light microscopy, and EPMA analysis, identified quartz, calcite, gehlenite, and akermanite as the primary mineral phases in bottom ash. They noted the amorphous phase constitutes approximately 50% of the ash and that the mineralogical composition is significantly influenced by incineration temperature. Wei et al. (2011) [21] utilized XRD, SEM/EDX, and XRF to confirm the primary oxide composition of bottom ash as SiO_2_ (40–50%), CaO (15–20%), and Al_2_O_3_ (8–12%), and revealed that gehlenite is extensively present as microcrystals embedded within the glass matrix. Building on this, Alam et al. (2019) [22] employed the XRPD Rietveld method combined with BCR sequential extraction to elucidate the association between heavy metals and specific mineral phases. They found Pb is predominantly associated with calcite and gehlenite, while Cr is mainly linked to gehlenite and the amorphous phase. De Matteis et al. (2023) [23] subsequently refined this approach by increasing acetic acid concentration to 5 M in the sequential extraction to ensure complete carbonate dissolution, and integrated XRPD, XRF, and ICP-MS for analysis. Their research indicated that fine particles have a high proportion of amorphous phases, with Pb and Zn mainly residing in the acid-soluble fraction. This results in a higher release potential than previously assessed by conventional methods, further clarifying the speciation and release mechanisms of potentially toxic elements in bottom ash. Similarly, Santos et al. (2013) [24] demonstrated via XRPD and XRF that accelerated carbonation treatment increases the content of calcite and gehlenite in bottom ash, effectively reducing the leaching rate of heavy metals. Collectively, these studies demonstrate that the formation and distribution of key mineral phases in bottom ash—particularly gehlenite—are decisive in determining the speciation and environmental behavior of heavy metals, thereby providing a critical mineralogical foundation for its safe disposal and resource recovery.

The physicochemical composition of MSWIBA varies considerably across regions, reflecting differences in waste management policies, energy sources, and consumption habits (Table 2). For instance, in some European countries such as Italy and the Netherlands, stringent waste segregation and pre-treatment practices often lead to bottom ash with relatively higher SiO_2_ content and lower heavy metal concentrations, making it more suitable for direct use in construction [25,26]. In contrast, in certain regions where municipal waste streams contain a larger proportion of food scraps and construction debris, the resulting bottom ash tends to exhibit more prominent CaO content and higher levels of soluble salts such as chlorides and sulfates. It should be noted that the composition of MSWIBA is strongly influenced by upstream waste sorting practices. Regions with established source separation programs generally produce bottom ash with higher SiO_2_ content and lower heavy metal concentrations, as recyclable materials such as glass, metals, and certain organics are diverted from the incineration stream prior to combustion. In contrast, regions where mixed waste incineration is more common typically yield bottom ash with greater compositional heterogeneity and higher contaminant loads [25,26]. These regional variations underscore the importance of tailoring valorization strategies to local material characteristics. Future research that systematically compares MSWIBA characteristics between industrial and agricultural regions could further elucidate the influence of waste source composition on ash properties.

Regulatory frameworks governing MSWIBA utilization vary across regions, reflecting differing approaches to waste management and environmental protection. In the European Union, MSWIBA is generally permitted for use in construction provided it meets leaching limit values specified in relevant directives, with end-of-waste criteria guiding its transition from waste to product [25,26]. In the United States, beneficial use of MSWIBA is encouraged under federal resource conservation policies, though state-level requirements vary considerably. In several Asian countries, including China and Japan, national standards have been established that set specific limits on heavy metal leaching from MSWIBA when used in construction materials, often mandating pretreatment such as aging or carbonation [27,28]. These regulatory differences underscore the need to tailor valorization strategies to local compliance requirements.

### 2.2. Speciation and Leaching Behavior of Heavy Metals

The environmental behavior of heavy metals in MSWIBA is essentially the result of the interaction between their speciation and the mineralogical matrix. Bottom ash is a multi-phase composite system, with more than 50% being an amorphous phase and the remainder consisting of crystalline minerals such as quartz, calcite, and gehlenite (Ca_2_Al_2_SiO_7_) [21]. These mineral phases control the speciation of heavy metals through complex molecular-scale interactions. Alam et al. (2019) [22] confirmed that Pb tends to form PbCO_3_ precipitates on the surface of calcite or embed into the gehlenite crystal lattice, while Cr^3+^ preferentially substitutes for Al^3+^ in gehlenite to form a solid solution. This lattice substitution mechanism renders Cr exceptionally stable under alkaline conditions. De Matteis et al. (2023) [23] further revealed that Zn in bottom ash mainly exists in the forms of Zn_5_(CO_3_)_2_(OH)_6_ and Zn(OH)_2_, with nearly all Zn being released in the acidic extraction step, and approximately 70% of the Zn content was extracted from bottom ash samples across different particle size fractions. Conversely, Pb mainly exists as carbonate (PbCO_3_) and Pb_3_O_4_ phases, which are unstable in highly acidic environments, leading to a significant difference in Pb leaching rates between larger and smaller particles. This microscopic mechanism explains the significant differences in the speciation of heavy metals. He and Kasina (2023) [2], using the BCR sequential extraction method, found that more than 80% of Cd and Zn, and more than 75% of Cu exist in the most easily mobile fraction, while Al and Fe are primarily associated with the reducible fraction, and Pb is completely correlated with the oxidizable fraction. This finding emphasizes that when utilizing MSWIBA, special attention must be paid to the leaching control of Cd, Cu, and Zn.

The leaching behavior of heavy metals is influenced by numerous environmental factors, with pH being a critical one. Yuganova and Putilina (2024) [4] note that pH and dissolved organic carbon (DOC) are key determinants of heavy metal leaching. Generally, the leaching of heavy metals increases under acidic or strongly alkaline conditions [30]. Zhang Hua et al. (2018) [31] systematically studied the non-linear impact of pH on leaching, finding that the leaching of Cd, Ni, and Zn begins to rise rapidly when pH < 8, while the leaching of Pb, Cu, Cr, and As increases significantly when pH < 4. Notably, as amphoteric metals, Pb and Zn also exhibit increased leaching under strongly alkaline conditions (pH > 11), though their maximum leaching concentrations under alkaline conditions are lower than under strongly acidic ones. The inherently high pH of bottom ash (approximately 11.28) creates an alkaline environment that inhibits metal leaching, a mechanism consistent with cement solidification as proposed by Xu Weixing (2017) [32], wherein the C-S-H gel surface formed by cement hydration stabilizes heavy metals as hydroxides or complexes via coordination bonds (e.g., ≡Si-O-Cd, ≡Al-O-Pb). Zhao et al. (2023) [33] further demonstrated that curing MSWIBA in semi-wet conditions promotes the solidification of heavy metals through C-S-H hydration and increased crystalline calcium carbonate. Tian et al. (2024) [34] found that when MSWI bottom ash is used as permeable road base material, its average pore size can encapsulate heavy metals, reducing their leachability. This dual physical-chemical fixation mechanism significantly decreases heavy metal mobility. Mineral phase transformation can alter the speciation environment of heavy metals; for instance, Santos et al. (2013) [24] achieved a 92% reduction in Pb leaching by increasing calcite content by 37% through accelerated carbonation. Similarly, Kokalj et al. (2025) [14] found carbonation significantly reduced barium and lead concentrations. Wehrung et al. (2024) [35] also observed that carbonation reduced leaching of several heavy metals but was less effective for anionic species like Sb and Cr, highlighting the complex interaction mechanisms between metals of different valences and mineral phases. Collectively, these findings indicate that the molecular-scale interactions at the heavy metal–mineral interface are the true determinants of environmental risk.

In addition to pH and mineral phase composition, aging or natural weathering significantly influences the long-term leaching behavior of heavy metals from MSWIBA. During stockpiling or environmental exposure, carbonation—the reaction of atmospheric CO_2_ with calcium-bearing phases—progressively alters the mineralogical matrix. Santos et al. [24] demonstrated that accelerated carbonation increased calcite content by 37% and reduced Pb leaching by 92%. Similarly, Nag et al. [36] reported that combined carbonation and pozzolanic reactions under optimized water-to-ash ratios achieved fixation rates exceeding 99% for Pb, Cr, and Cu within 60 days. However, the effects of aging are not uniformly beneficial; Wehrung et al. [35] observed that while carbonation reduced leaching of several cationic heavy metals, it was less effective for anionic species such as Sb and Cr. These findings highlight that natural weathering processes can both stabilize and, in some cases, selectively mobilize certain elements, underscoring the need to consider aging effects when evaluating the long-term environmental safety of MSWIBA in outdoor applications.

### 2.3. Seasonal Variation and Particle Size Effects

The composition and heavy metal content of MSWIBA are not static but vary significantly due to factors like incineration processes and seasonal changes.

Particle size distribution is a critical determinant of both the composition and potential applications of bottom ash. Beikmohammadi et al. (2023) [11] analyzed the particle size distribution of incineration bottom ash in Tehran and found that concentrations of heavy metals, including zinc, copper, barium, lead, chromium, nickel, tin, vanadium, arsenic, and antimony, were higher in particles smaller than 4 mm. In contrast, precious metals like gold and silver were significantly enriched in the fine particles (<0.5 mm). This finding offers a theoretical basis for recovering valuable metals through physical separation and underscores the need for pre-treatment before bottom ash can be used in civil or municipal projects. Ghani et al. (2023) [12] corroborated this with XRF analysis, confirming high levels of major elements like calcium, iron, aluminum, magnesium, and sodium in the bottom ash. They also noted enrichment factors (EF) greater than 30 for zinc, copper, and lead, and an extraction degree (DE) exceeding 80% for elements in the fine-particle fraction, further substantiating the enrichment of heavy metals in smaller particles. Furthermore, Valentim et al. (2024) [37] performed a geochemical analysis of Portuguese incinerator residues, indicating that most trace elements concentrate in the fine-particle fraction. The study also found that potassium concentrations surpassed recovery thresholds, suggesting that its concentration and recovery could be achieved through simple washing.

While research on particle size distribution is more established, systematic studies on the seasonal variations in bottom ash composition are limited, impeding a comprehensive understanding of its characteristics. Existing studies suggest that the elemental composition of MSWIBA does fluctuate seasonally, a phenomenon primarily driven by seasonal variations in raw waste composition, climatic shifts, and cyclical changes in consumer behavior. Chuchro and Bielowicz (2025) [10] conducted an exploratory analysis of seasonal variations in Al, Cu, Fe, and Zn concentrations, finding higher metal levels during colder months, potentially linked to an increased influx of electronic waste and metal packaging in winter. Although this analysis was limited to one year and four metals, it serves as a vital reference for understanding seasonal dynamics. Their other research observed seasonal patterns in Ba and Sr content, as well as in the distribution of Al and heavy rare earth elements, highlighting the importance of long-term monitoring and seasonal analysis for optimizing resource recovery and evaluating environmental risks [38]. Moreover, Denafas et al. (2014) [39] demonstrated in a study of four Eastern European cities that municipal solid waste composition can vary by over 8% across seasons, a variation that directly impacts the properties of the resulting incineration bottom ash. Collectively, this body of research reveals the complexity and heterogeneity of bottom ash’s physicochemical composition, providing foundational data for developing strategies in both high-value utilization and risk management.

## 3. High-Value Utilization of MSWIBA

As a secondary resource generated in large quantities, the high-value utilization of MSWIBA is crucial for developing a circular economy and achieving sustainable waste management. Current research primarily centers on two complementary valorization routes: the recovery of valuable metals (including ferrous, non-ferrous, and precious metals) and the utilization of MSWIBA as a substitute for conventional building materials. These two approaches can be implemented either sequentially—where metals are first recovered and the residual material is then used in construction—or in parallel, depending on local economic conditions, market demands, processing capabilities, and regulatory frameworks [25,26,40]. Together, these routes not only conserve natural resources and reduce the burden on landfills but also yield substantial economic and environmental advantages.

### 3.1. Recovery of Valuable Metals

#### 3.1.1. Recycling of Ferrous and Non-Ferrous Metals

MSWIBA generally contains about 5–10% ferrous metals (mainly iron) and 1–3% non-ferrous metals (such as aluminum and copper). Conventional physical recycling techniques rely on the disparity in the physical properties of these metals: magnetic separation is effective for recovering ferrous metals, while eddy current sorting leverages the electrical conductivity of non-ferrous metals to achieve separation. Adhiwiguna et al. (2025) [41] further integrated magnetic separation, eddy current sorting, and density sorting to establish an enhanced value-added treatment process. By synergistically exploiting multiple physical property differences, this approach not only improved the efficiency and enrichment of metal separation but also yielded mineral components of higher purity.

Particle size distribution is a critical factor in guiding the selection of recycling processes. Research by Śyc and Hykš (2025) [42] revealed that recoverable metals exhibit enrichment in specific particle size ranges: larger metal fragments, including iron, aluminum, and copper, are predominantly found in coarse particles larger than 2 mm. This finding supports a physical sorting process that does not require extensive comminution. Conversely, Ghani et al. (2023) [12] indicated that metals such as Zn, Cu, and Pb display greater chemical leachability in fine particles measuring 0.5–1 mm, with extraction rates exceeding 80%. This characteristic is closely associated with the high specific surface area of fine particles and the predominance of metals in easily soluble phases.

Based on these particle size characteristics, a tiered recovery strategy can be formulated: For coarse particles (>2 mm) rich in metals, physical methods like magnetic and eddy current sorting are appropriate for efficient, direct recovery. In contrast, for fine particles with high metal reactivity, chemical or electrochemical methods are more suitable for in-depth extraction. For instance, the sequential electrochemical extraction and chemical precipitation process developed by Zhang et al. (2024) [1] utilizes the varying reduction potentials of metal ions to achieve the efficient, selective recovery of multiple metals, with both recovery rates and purity for all elements surpassing 90%. Furthermore, Keber et al. (2023) [43] optimized a flotation process for copper in fine-grained materials by employing the collector AERO MX-5160 in conjunction with alginic acid as an inhibitor, enabling the highly selective separation of copper.

#### 3.1.2. Rare and Precious Metal Recovery

MSWIBA also contains rare metals (such as rare earth elements) and precious metals (such as gold and silver), whose recovery potential is garnering increasing attention. Research indicates that the speciation of these elements is closely correlated with their particle size distribution. Muchova et al. (2009) [44] were among the first to confirm that bottom ash is rich in precious metals like gold and silver, noting that their sources are particle size-dependent: precious metals in fine particles (<2 mm) primarily originate from Waste Electrical and Electronic Equipment (WEEE), whereas those in larger particles (2–6 mm) mostly derive from jewelry waste. Beikmohammadi et al. (2023) [11] further discovered that gold, silver, and rare earth elements (e.g., Ce, Nd, La, Y) are significantly enriched in even finer particles (<0.5 mm).

Addressing these particle size characteristics, research has progressively shifted toward developing efficient and environmentally benign extraction and stabilization methods. In the realm of organic acid extraction, Cao et al. (2025) [45] systematically compared the performance of four bio-based organic acids. They found citric acid (CA) to be highly efficient for extracting elements such as Mn, Pb, Co, Cd, Zn, and Ni (>50%), a process primarily driven by proton-promoted dissolution and ligand complexation. Conversely, oxalic acid (OA) proved more advantageous for elements like Sn, Sb, Mo, and Cr (>60%), functioning by forming insoluble calcium oxalate with calcium minerals in the ash, thereby co-precipitating and immobilizing heavy metals like Pb, Zn, and Sr. Following treatment with CA or OA, supplemented by Ca(OH)_2_ neutralization and washing, the leaching toxicity of the bottom ash was drastically reduced (removal rate >99.9%), rendering it compliant with standards for use in construction materials. For resource quantification and enrichment, Morf et al. (2013) [46] utilized material flow analysis to demonstrate that the Thermo-Re^®^ thermal sorting process can enrich gold in the non-ferrous metal fraction below 5 mm by a factor of nearly 100, establishing a quantitative basis for large-scale recovery.

To further enable the efficient, selective recovery of rare earth elements and the recycling of adsorbents, Li et al. (2024) [47] synthesized a phosphate-functionalized magnetic mesoporous SiO_2_ adsorbent (MMS-PP). Leveraging its magnetic core, high-specific-area mesopores, and specific phosphate functional groups, this material achieved the efficient adsorption of rare earth ions from leachates, followed by the magnetic separation and recovery of the adsorbent itself. To holistically integrate resource recovery with waste management in an environmentally sustainable manner, Wen et al. (2024) [13] proposed a three-step system: “citric acid leaching-oxalic acid precipitation-residue zeolite conversion.” This system operates by first using citric acid to chelate and leach rare earths at pH 2.0 (achieving > 80% recovery). Subsequently, rare earths are concentrated 7–12 fold via oxalate precipitation. Finally, the leaching residue is hydrothermally converted into zeolite, simultaneously achieving an approximate 80% reduction in solid mass and the stabilization of heavy metals. This integrated system offers a practical, low-input, and low-environmental-impact method for recovering rare earth elements from MSWI bottom ash and managing the residual waste.

#### 3.1.3. Bioleaching and Electrochemical Separation

Beyond physical separation and chemical extraction, emerging technologies such as bioleaching and electrochemical separation offer new methodologies for recovering metals from MSWIBA, particularly for the removal and valorization of heavy metals. While some research has targeted incineration fly ash, the underlying principles are applicable to bottom ash as well.

Bioleaching is an environmentally promising technology that utilizes metabolic products from microorganisms to dissolve metals. In process development, Mäkinen et al. (2019) [48] conducted heap leaching experiments to assess the technology’s feasibility at an engineering scale. Their findings confirmed that iron/sulfur-oxidizing bacteria in a sulfate medium could achieve leaching efficiencies of 18–53% for zinc and 6–44% for copper. Gomes et al. (2020) [49] employed a mixed culture of acidophilic bacteria to bioleach fly ash, achieving removal rates exceeding 90% for metals like Zn and Cu. This process primarily involves acidophilic bacteria oxidizing sulfur or iron to generate sulfuric acid and ferric ions, which dissolve metals through acid dissolution and oxidation. At a mechanistic level, Kucera et al. (2025) [50] used diaPASEF proteomics to reveal how *Acidithiobacillus ferrooxidans* adapts to heavy metal stress in incineration residue environments by upregulating membrane transport proteins, metal efflux pumps, and electron transfer proteins, with this molecular response being most pronounced at moderate residue concentrations. In addition to acidophiles, *Pseudomonas aeruginosa* PAO1 has been used for heavy metal bioleaching. Research by Biswal et al. (2020) [51] indicated that while microbial activity can enhance the dissolution of heavy metals like Cu and As, subsequent geopolymerization can significantly reduce their leachability through physical encapsulation, adsorption, or chemical bonding, highlighting its potential for heavy metal stabilization.

To enhance the selectivity and efficiency of metal separation, electrochemical separation has been integrated with bioleaching in coupled processes. This method employs a direct electric field to drive the directional migration and enrichment of metal ions via mechanisms like electromigration and electroosmotic flow. Although studies often use fly ash, the technical principles are relevant for bottom ash. For instance, Appiah et al. (2024) [52] demonstrated that electrodialysis could remove 94–100% of heavy metals like Cd, Cu, Zn, and Co from fly ash, with significant reductions in acid consumption achievable through electrolyte system optimization, underscoring its benefits in chemical volume reduction. To further improve metal recovery in bioleaching systems, research has focused on electro-biological hybrid systems. Narenkumar et al. (2023) [53] combined bioleaching by acidophilic sulfur-oxidizing bacteria with electrokinetic remediation. This approach used microbial acid production to dissolve metals and an electric field to drive their migration, increasing removal rates of alkali and alkaline earth metals (K, Na, Ca, Mg) to 85–92%, though it also identified selectivity limitations for metals such as Pb. Gomes et al. (2020) [54] developed a microbial fuel cell to demonstrate that the synergy between electromigration and microbial acid generation at low voltages greatly enhances the migration and enrichment of metals like Cu and Cr, providing a mechanistic explanation for how electrochemical processes can boost the separation efficiency in bioleaching.

Current research reveals that a single technical approach has limitations when treating incineration ash and slag with complex compositions. Kinnunen and Hedrich (2023) [55] suggest that future advancements hinge on the systemic integration of processes, such as employing bioleaching as a preliminary stage for metal activation and dissolution, coupling it with electrochemical or membrane separation techniques for the selective recovery of target metals, and stabilizing the final residue. This tiered integration strategy is poised to enhance resource recovery efficiency while reducing the overall environmental footprint of the process, thereby accelerating the practical application of these technologies in bottom ash management.

For the recovery of valuable metals, existing technical routes are generally hampered by the trade-off between recovery efficiency and processing costs. While physical separation methods achieve high recovery rates for metals like iron and aluminum in coarse particles, their effectiveness in separating fine particles (<0.5 mm) containing concentrated zinc, copper, precious metals, and rare earth elements is limited, which results in resource loss [11,12,41]. Although hydrometallurgical or electrochemical methods can efficiently extract valuable metals from fine particles, they involve complex processes, high reagent consumption, and the generation of secondary wastewater, with their economic viability being highly sensitive to fluctuations in metal market prices [1,45]. Bioleaching presents an environmentally friendly alternative, but challenges such as slow reaction rates, lengthy processing times, and susceptibility of microbial activity to substrate inhibition have prevented its large-scale industrial implementation to date [48,50,53]. Consequently, there is a pressing need to develop a comprehensive and integrated technical system for metal recovery and hierarchical resource utilization that encompasses the full spectrum of particle sizes, offers high selectivity, imposes a low environmental burden, and maintains economic feasibility.

In conclusion, to elucidate the core characteristics and practical limitations of each technology and to facilitate the optimization and selection of future process routes, the principal recovery technologies are summarized and contrasted in Table 3.

### 3.2. Utilization as Construction Materials

#### 3.2.1. Cement-Based Materials (Concrete, Mortar, Cementitious Materials)

The incorporation of MSWIBA into cement-based materials represents one of the most extensively investigated methods for resource recovery. MSWIBA can serve as a cement substitute, aggregate, or filler, though its efficacy and limitations are contingent upon the dosage and pre-treatment methods employed.

Utilized as a cement replacement, MSWIBA’s pozzolanic activity enables it to partially substitute cement, thereby reducing carbon emissions associated with cement production. Vilarinho et al. (2023) [5] observed that replacing 15% of ordinary Portland cement with bottom ash resulted in a mortar compressive strength of 9.3 MPa, which was deemed suitable for low-strength walls. Wijesekara et al. (2024) [56] noted that sintered bottom ash and vitrified ash could preserve mortar strength at 10% and 25% substitution levels, with the sintered variant demonstrating superior potential due to its alkalinity and pozzolanic reactivity. Czop and Łaźniewska-Piekarczyk (2020) [6] assessed the viability of using MSWI slag to replace 30% of cement, achieving a 28-day compressive strength of 32.0 MPa and a flexural strength of 4.0 MPa in the resulting mortar, with leachate tests indicating minimal environmental impact. Reddy and Kumar (2025) [9] reported that MSWI bottom ash, even at a 20% replacement ratio, did not adversely affect the mechanical properties of concrete and could enhance hydration and freeze–thaw resistance. Nevertheless, Malaiškienė et al. (2023) [57] found that while ground MSWIBA, acting as a micro-filler, improved mortar workability, enhancing mechanical properties required supplementary additives like metakaolin. Bielen et al. (2025) [58] indicated that a 10% MSWIBA replacement was more effective than 20%, and when combined with ground slag, the mixture achieved strength comparable to conventional cement at a 120-day age. Altaher et al. (2025) [59] optimized the performance of MSWI ash mortar by incorporating 10% rice husk ash, which improved mechanical properties while reducing water absorption.

When used as an aggregate or filler, MSWIBA can replace natural aggregates in concrete or mortar or serve as a filler to refine the microstructure and increase density. Vilarinho et al. (2023) [5] reported that substituting natural aggregates with MSWIBA generally led to a decline in mortar performance, a phenomenon linked to the irregular shape, rough surface, and high water absorption of bottom ash particles, or insufficient chemical reactivity. However, Minane et al. (2025) [60] demonstrated that when blast furnace slag cement was used, mortar with a 50% aggregate replacement could attain a 50 MPa compressive strength at 180 days, with no health risks detected in leachate tests, underscoring the critical influence of cement type on the effectiveness of bottom ash utilization. Satkunarasa and Baskaran (2023) [61] further corroborated the feasibility of a 50–56% bottom ash replacement in residential construction, though this was accompanied by reduced aggregate density, diminished strength and durability, and heightened water absorption. Thomas and Ślosarczyk (2023) [62] similarly found that a 50 vol.% aggregate replacement significantly impaired mortar workability and water absorption and reduced mechanical properties, whereas the use of high-strength cement could enhance compressive strength, freeze–thaw resistance, and abrasion resistance. Furthermore, MSWIBA fine powder, when used as a filler, can improve the microstructure of cementitious materials. Hanghang et al. (2024) [63] investigated MSWI ash micro-powder as a concrete admixture, identifying its potential hydraulicity but noting its slow hydration process; increasing the dosage was found to delay hydration and degrade mechanical properties.

The mechanisms behind the performance deterioration from high dosages of MSWIBA can be attributed to several factors: the porous structure of unburned carbon absorbs water and hydration products, thereby retarding the hydration process and weakening the interfacial bond [64]; soluble salts (such as Na_2_O, K_2_O, and Cl^−^) and residual unburned organic matter interfere with the hydration reactions [65]; aluminum metal reacts in an alkaline environment to produce hydrogen gas, creating pores and micro-cracks [64,66]; certain silica-bearing minerals induce an alkali–silica reaction, resulting in expansion and cracking [64]; and chloride and sulfate salts trigger secondary reactions that destabilize the C-S-H gel [64,67]. Additionally, the inherent physical defects (e.g., irregular particle shapes and high porosity) and chemically inert components of the bottom ash itself further worsen the performance degradation at high dosages [67,68].

Alkali-activated cementitious materials, through geopolymer technology, represent an innovative approach to creating a binding matrix by activating the silicon and aluminum constituents within MSWIBA using strong alkalis. Liu et al. (2023) [69] designed an alkali-activated system based on a Ca/Si ratio for the synergistic treatment of fly ash and bottom ash, which yielded a 56-day compressive strength of 8.8 MPa and achieved effective heavy metal immobilization. Tamošaitis and Vaičiukynienė (2025) [70] employed a triple alkali activator to stimulate bottom ash at a lower pH (<12.5), achieving a compressive strength of 20 MPa. Deng et al. (2024) [71] observed that in a sodium silicate-activated MSWIBA-slag composite system, the porosity increases with higher bottom ash content, while excessive alkalinity inhibits both hydration and strength development. Tamošaitis et al. (2025) [72] utilized a low water-to-binder ratio vibration compaction technique, in combination with metakaolin, to produce alkali-activated concrete with a compressive strength of 40.0 MPa. Setlak et al. (2023) [73] synthesized a geopolymer binder from silicon-aluminum-rich waste through alkali activation, achieving a strength surpassing 57 MPa. Naqi et al. (2022) [74] highlighted that high alkalinity, a high SiO_2_ content, and a low water-to-binder ratio are beneficial for enhancing the early hydration and strength development of the alkali-activated system. Collectively, these studies demonstrate that alkali activation technology offers a promising avenue for the valorization and heavy metal stabilization of MSWIBA.

Beyond mechanical performance, the potential environmental and human health risks associated with incorporating MSWIBA into cement-based materials are of paramount concern. As detailed in Section 2.2, the speciation and leaching behavior of heavy metals (e.g., Pb, Zn, Cu) in MSWIBA are governed by complex interactions with mineral phases and are highly sensitive to pH and environmental conditions [4,22,24].Under standard leaching tests, several studies have reported that mortars containing MSWIBA comply with regulatory limits, indicating acceptable short-term environmental compatibility [6,60]. However, the long-term stability of these solidified products under real-world conditions—such as acid rain infiltration, carbonation, freeze–thaw cycles, and mechanical loading—remains inadequately characterized [4,65]. For example, carbonation can lower the internal pH of the matrix, potentially remobilizing amphoteric metals like Pb and Zn, as discussed in Section 4.1 [14,35]. Additionally, the generation of fine dust during the crushing and mixing of MSWIBA-containing materials poses an inhalation risk to workers, highlighting the need for appropriate dust control measures. These considerations underscore that sustainable utilization of MSWIBA in construction requires not only performance optimization but also rigorous long-term environmental impact assessments, standardized quality control protocols, and life-cycle analysis frameworks that account for both resource efficiency and risk mitigation.

#### 3.2.2. Utilization as Construction Materials/Road Engineering Materials

In road engineering, the resource utilization of Municipal Solid Waste Incinerator Bottom Ash (MSWIBA) demonstrates varied performance characteristics. Xu et al. [75] employed bottom ash as a direct replacement for limestone in asphalt mixtures. Their research indicated that while the mixtures exhibited adequate resistance to high-temperature deformation, the fatigue life decreased by more than 42%. This reduction was primarily due to the smooth, near-spherical morphology of the bottom ash particles and their weak adhesion to asphalt, which resulted in insufficient interfacial bonding. Consequently, these areas became vulnerable points for the initiation of fatigue cracks under repeated loading. To mitigate this issue, Park et al. (2023) [76] introduced waste asphalt shingles in a composite with bottom ash. The aged asphalt present in the shingles compensated for the bottom ash’s tendency to absorb asphalt, while the fibers and rigid particles reinforced the mixture’s skeletal structure, thereby enhancing both high-temperature stability and fatigue resistance [77]. Sun et al. (2025) [8], through analysis using the Mechanistic-Empirical Pavement Design Guide, predicted that a well-designed asphalt pavement containing bottom ash, though somewhat less resistant to rutting, shows significant potential for resisting fatigue cracking and maintaining long-term smoothness. This may be attributed to the improved interfacial bonding promoted by the surface chemistry of the bottom ash and its mild pozzolanic reaction. Within cement-stabilized base courses, Węgliński and Martysz (2024) [7] observed that incorporating 30–45% bottom ash improved both early strength and frost resistance. This is because components like CaO and SiO_2_ participate in hydration reactions, producing additional cementitious products, and the porous structure of the bottom ash helps to mitigate stresses from freeze–thaw cycles. Furthermore, Tian et al. [34] confirmed the viability of using bottom ash as a permeable subgrade material. The specific particle gradation of the bottom ash creates a connected pore structure, which provides excellent permeability and the capacity to adsorb pollutants from rainwater.

#### 3.2.3. Bricks, Tiles, and Other Construction Products

The variations in performance between bricks and other construction products are primarily attributable to the distinct ways in which different manufacturing processes regulate the microstructure.

In the traditional sintering method, material properties are governed by high-temperature phase transitions and the evolution of porosity. For instance, Kizinievič et al. [78] substituted 5–15% of the clay with bottom ash, which was then sintered at 1000 °C. As the proportion of bottom ash increased, elements such as calcium and iron formed low-temperature eutectic phases, which helped maintain a compressive strength of 32–44 MPa. However, this also led to an increase in open porosity and water absorption, consequently reducing frost resistance. Pitak et al. [79] conducted a systematic investigation into the synergistic effects of sintering temperature and ash content. They found that at ash contents below 20%, the pores generated from the decomposition of CaCO_3_ in the bottom ash did not create an interconnected network. This resulted in a lower thermal conductivity that was still sufficient for general environmental applications. However, once this threshold was exceeded, the pore connectivity increased substantially, leading to a degradation in durability.

An alternative approach is the chemical activation pathway, which develops strength through cementitious reactions at ambient temperatures. Zhou and Wang [27] employed a two-step process involving pre-treatment with NaOH to remove aluminum, followed by activation with Ca(OH)_2_. This method not only eliminated the risk of expansion due to aluminum but also promoted the dissolution of silico-aluminates, forming a dense C-(A)-S-H gel. The resulting bricks achieved a compressive strength of 11.22 MPa (equivalent to the MU10 grade) with a 30.6–44.2% reduction in energy consumption compared to traditional sintered bricks. Furthermore, compound and modified systems have expanded the dimensions of performance tuning. Alam et al. [29] leveraged cement hydration to generate C-S-H gel from the silico-aluminous components in bottom ash, producing lightweight, cost-effective bricks with a strength of 7.13 MPa. Similarly, Tessema and Fantahun (2023) [80] used molten thermoplastic plastic as a binding phase to coat bottom ash and textile sludge, creating high-strength, eco-friendly bricks with a mere 1.3% water absorption rate. The Naganathan team [81] introduced fly ash to optimize the particle gradation, enabling the achievement of self-compaction under a low water-to-binder ratio. This produced non-fired bricks with a dense structure and excellent mechanical and fire-resistant properties.

A high-temperature phase transformation route offers a significant performance improvement by completely reconstructing the material’s phase composition. Acampora et al. (2025) [82] utilized a vitrification process exceeding 1000 °C. The bottom ash was melted, and its crystallization was carefully controlled to form a microstructure composed of a glass matrix reinforced by crystalline phases. This process yielded glass-ceramic bricks with a remarkable flexural strength of 60 MPa, combined with superior durability and aesthetic qualities.

The primary challenge in utilizing MSWIBA as a construction material is overcoming its inherent deficiencies to ensure the performance and reliability of the final product. In cement-based composites, the addition of bottom ash typically causes a decline in mechanical properties, particularly at high dosages, where compressive strength and long-term durability are significantly reduced. This is largely due to the irregular particle morphology of MSWIBA, its high water absorption, and the presence of impurities such as unburned carbon, chlorides, and reactive aluminum. These factors interfere with cement hydration and disrupt the interfacial transition zone between aggregates and the paste, thereby compromising the material’s overall integrity [5,61,63,64,65,66,67,68,83]. While alkali-activation technology is more effective at immobilizing heavy metals and activating pozzolanic potential, its reaction process is highly sensitive to the chemical composition of the raw materials (e.g., the SiO_2_/Al_2_O_3_/CaO ratio) and the specific activator system. This results in a narrow processing window, posing challenges for maintaining homogeneity and quality consistency during large-scale production [69,70]. In road construction, the application of MSWIBA as a filler in asphalt mixtures is hindered by its poor adhesion to asphalt, which can reduce the mixture’s fatigue resistance. When used in stabilized inorganic bases for roads, a delicate balance must be struck between achieving sufficient material strength and ensuring long-term volume stability [75,76].

Despite these performance advantages, the environmental safety of bricks and tiles manufactured with MSWIBA must be carefully evaluated. The primary concerns, similar to those for cement-based materials, are the potential leaching of heavy metals over the product’s lifetime and the risk of dust inhalation during manufacturing. Studies on sintered and alkali-activated bricks have shown that high-temperature or chemical stabilization processes can effectively immobilize heavy metals, resulting in leachate concentrations below regulatory thresholds [27,78]. However, the durability of this immobilization under the combined effects of weathering, freeze–thaw cycles, and long-term use requires further validation. Therefore, comprehensive life cycle assessments that include both mechanical performance and long-term environmental impact are essential for validating the sustainability of MSWIBA-based masonry products.

In conclusion, the application methods, performance profiles, and limitations of MSWIBA in various building materials differ considerably. For a clear comparative analysis, the key information from the utilization pathways discussed above is summarized in Table 4.

## 4. Risk Control Technologies for MSWIBA

The high-value application of MSWIBA is contingent upon rigorous environmental risk control, with the leaching potential of heavy metals being the foremost concern. Consequently, the development and implementation of effective risk mitigation technologies are paramount. Based on their underlying principles, current technologies are primarily classified into two categories: stabilization and separation/removal. These categories will be systematically examined below.

### 4.1. Solidification/Stabilization (S/S) Technology

Solidification/Stabilization (S/S) technology mitigates the mobility and bioavailability of heavy metals via physical encapsulation or chemical transformation, ensuring their long-term stability within the environment. The predominant solidification technologies currently employed in engineering practices fall into four categories: cement-based, alkali-activated, thermal treatment, and carbonation. Each is suited to specific applications according to its respective mechanism.

Cement-based solidification stands as one of the most prevalent and economical S/S methods. This technique combines MSWIBA with cement, water, and supplementary admixtures to create a dense, solidified matrix. Heavy metals are immobilized either by being entrapped within hydration products (e.g., C-S-H gel) or by adsorption and precipitation mechanisms. Liu et al. (2023) [69] designed an alkali-activated cementitious material based on the Ca/Si ratio, achieving the synergistic treatment of MSWI fly ash and bottom ash. This process immobilized over 98% of heavy metals (Cd, Cr, Cu, Ni, Pb, Zn), with leaching concentrations below the limits stipulated by Chinese national standards. Zaleska et al. (2023) [84] demonstrated that the incorporation of incineration ash enhanced the flexural and compressive strength of the material and markedly decreased the leaching of Pb and Zn. Valizadeh et al. (2024) [85] assessed solidification protocols for cement production by analyzing ash from Tehran and Nowshahr, finding that a blend containing 20% ash and 10% zeolite could limit metal release to 37 g/day, suggesting ash solidification serves as a viable complementary management strategy for waste incineration. Nevertheless, cement-based solidification presents certain limitations; chlorides and sulfates present in MSWIBA can impede cement hydration, compromising long-term stability, and the process typically results in an increased final volume.

Alkali-activated solidification offers a dual benefit, serving as both a pathway for MSWIBA valorization and a highly effective means of heavy metal stabilization. Under strong alkaline conditions, the silico-aluminous components of MSWIBA form a three-dimensional network gel structure. Heavy metal ions are subsequently stabilized through mechanisms such as physical encapsulation, ion exchange, and surface complexation. Liu et al. (2023) [69] verified the efficacy of alkali-activated cementitious materials in stabilizing heavy metals from MSWI fly ash and bottom ash. Deng et al. (2024) [71] further elaborated that a sodium silicate-activated MSWIBA-GGBS system primarily generates N-A-S-H and C-A-S-H gels, which play a crucial role in the immobilization of heavy metals.4 Risk Control Technologies for MSWIBA.

Heat treatment technologies, such as sintering and vitrification, reconstruct the mineral phases of MSWIBA at high temperatures, immobilizing heavy metals within an amorphous phase or stable crystal lattices and thereby significantly reducing their leaching potential. He et al. (2025) [86] noted that while heat treatment can achieve volume reduction and the fixation or recovery of heavy metals, it is a high-energy-consumption process. Sirico et al. (2024) [87] demonstrated that substituting 20% of cement with vitrified MSWI bottom ash can enhance concrete sustainability. Kang et al. (2025) [88] evaluated plasma melting technology for fly ash treatment, finding that the resulting slag had a pH of 9.9 with no toxic elements detected, and complete removal of chlorides, fluorides, and sulfates, meeting soil environmental standards and highlighting the potential of heat treatment for complete detoxification. The cold sintering process (CSP) investigated by Liao et al. (2023) [89] provides a novel method for stabilizing heavy metals at low temperatures. Importantly, heat treatment—particularly vitrification—can simultaneously stabilize heavy metals and convert bottom ash into an active precursor rich in an amorphous phase, serving as high-quality feedstock for alkali-activated cementitious materials (geopolymers), thus fostering a deep integration of “stabilization” and “valorization.”

Carbonation treatment leverages CO_2_ to react with the calcium components in MSWIBA, forming calcium carbonate and precipitating and fixing heavy metals. Kokalj et al. (2025) [14] showed that CO_2_ gas streams, water spray, and their combinations can substantially reduce the leaching concentrations of Ba and Pb. Wehrung et al. (2024) [35] found that carbonation suppresses the leaching of most heavy metals but has limited effectiveness for fixing anionic species such as Sb and Cr. Nag et al. (2025) [36], using an “ash-to-ash treatment method” (AATM) to optimize the water-to-bottom ash ratio, discovered that carbonation and pozzolanic reactions—acting in concert to lower pH, form calcite, and generate new minerals and C-S-H gel—achieved fixation rates exceeding 99% for Pb, Cr, and Cu within 60 days.

A central common challenge for various risk control technologies is the inadequate validation of their long-term environmental safety. For instance, Solidification/Stabilization (S/S) technology effectively suppresses heavy metal leaching in the short term, but its durability and stability under the combined long-term impact of environmental factors like acid rain, freeze–thaw cycles, and carbonation remain uncertain. Specifically, cement-based solidified bodies may experience heavy metal re-mobilization due to phase changes in hydration products. The long-term phase evolution of alkali-activated materials is not yet fully understood. While heat treatment offers significant immobilization, it is energy-intensive. Carbonation is limited in its ability to fix anionic heavy metals like Sb and Cr, and the stability of its products in acidic environments requires further verification [14,35,86]. Currently, there is a lack of accelerated aging test methods capable of accurately simulating material performance over several decades and reliable long-term performance prediction models, leaving a gap in the fundamental basis for assessing the environmental safety of solidified materials throughout their life cycle.

To define the applicable conditions for various solidification technologies, this section synthesizes their core mechanisms, performance characteristics, applicable conditions, and typical applications into Table 5, intending to provide a reference for engineering decision-making.

### 4.2. Pretreatment Technologies

Besides stabilizing heavy metals within a solid matrix, a critical alternative is pretreatment technology. This method focuses on reducing or eliminating harmful substances—such as chlorides and heavy metals—from MSWIBA before its use, through physical or chemical processes, to enhance its safety and viability for application.

Water washing is the most straightforward pretreatment technique, primarily targeting soluble salts, especially chlorides, in MSWIBA. Lu et al. (2020) [90], through simulated column experiments, demonstrated that slow leaching effectively reduces the soluble chloride concentration in bottom ash. Treatments of fine particles (<0.5 mm, as discussed in Section 2.3) and coarse particles (>2 mm) resulted in reductions of 75.0% and 68.4%, respectively, though they noted the method’s significant water consumption. Their subsequent research [91] further confirmed that water washing substantially decreases the leaching concentrations of heavy metals like Cu, Zn, and Ni. However, the water washing process is complex, and its efficacy is influenced by numerous factors. A study by Sun and Yi (2020) [92] elucidated the dynamic evolution of pH during washing and its crucial role in regulating heavy metal removal efficiency: the pH of the washing solution rises rapidly due to the dissolution of alkaline substances, precipitating and removing metal hydroxides like Pb, Zn, and Ni. As washing time increases, however, a pH decline can cause concentrations of Cd, Cu, and Cr to rebound. Consequently, precise control of washing duration and real-time pH is essential for optimizing the process. This indicates that simple water washing is not a universally applicable solution, and its operational parameters must be meticulously tailored to the specific target pollutants. Moreover, the limitations of water washing have received considerable attention. Li et al. (2025) [93] noted that even multi-stage water washing achieves relatively low removal efficiencies for heavy metals (10.28–19.38%) and may generate wastewater with hazardous levels of heavy metals, creating a risk of secondary pollution. Furthermore, a life cycle assessment by Peceño et al. (2024) [94] suggested that the water washing process can increase environmental burdens, such as marine eutrophication and human toxicity.

To overcome the drawbacks of water washing and achieve more thorough removal or resource recovery of pollutants, researchers have developed more powerful chemical pretreatment methods. Sun and Yi (2021) [95] further investigated acid washing, finding that nitric acid effectively removed Zn, Cu, and Ni (with efficiencies of 62–76%), but was less effective for Pb, Cr, and Cd. Subsequent stabilization with MgO successfully suppressed heavy metal leaching, allowing the material to meet stricter disposal standards. This highlights the potential of combined “chemical leaching + stabilization” processes. A more innovative approach involves converting bottom ash into high-value functional materials. Luo et al. (2019) [96] utilized a hydrothermal method to treat bottom ash and, with the aid of humic acid, successfully synthesized tobermorite. This material exhibited an adsorption capacity for Cu(II) in aqueous solutions of up to 270.3 mg/g, representing a significant upgrade from mere waste treatment to the creation of functional materials. Lin et al. (2025) [15] subsequently discovered that combining alkali fusion with hydrothermal technology could reduce the soluble chloride content in bottom ash to below 0.02%, achieving near 100% removal efficiency, while simultaneously synthesizing high-quality zeolites (such as ZSM-23). Ultimately, the practical value of any pretreatment technology must be validated through engineering applications. Kim et al. (2023) [97] conducted a systematic evaluation of how pretreating bottom ash with different water qualities (tap, deionized, saline, and seawater) affects the performance of bottom-ash-mortar systems. They found that while pretreatment generally improved the long-term strength of the mortar, different methods impacted the system’s chloride ion content, hydration products, and drying shrinkage performance in varying ways. This provides a crucial basis for selecting an appropriate pretreatment scheme tailored to specific engineering requirements.

Current pretreatment and end-of-pipe technologies face a dual challenge: a high risk of secondary pollution and the difficulty of forming a complete technical closed loop. The key to overcoming this challenge is to systematically integrate and coordinate pretreatment technologies with subsequent risk control or resource recovery stages, according to their core objectives and effectiveness. For instance, while water washing pretreatment can effectively remove soluble chlorides and some easily soluble heavy metals to create favorable conditions for cement-based solidification or carbonation, the process produces wastewater with high concentrations of salt and heavy metals. This wastewater is expensive to treat and can cause secondary water pollution if not handled properly [93,94]. Acid washing pretreatment can deeply leach heavy metals like Zn, Cu, and Ni, significantly reducing the pollutant load of the bottom ash. This makes the treated material more suitable for high-temperature processes like sintering or vitrification, which require high thermal stability, or for high-purity alkali-activated systems. However, this method uses highly corrosive chemicals, leading to high operational risks, and the resulting liquid waste and residues require further treatment to be rendered harmless. A fully green, closed-loop process has not yet been established [15,98]. Alkali fusion–hydrothermal combined treatment not only efficiently removes chlorine but also transforms bottom ash into a precursor rich in reactive silica and alumina components. This serves as a high-quality feedstock for alkali-activated solidification, and the synthesized products, such as zeolites, can be directly used in adsorption. However, this process has relatively high energy consumption. Regarding adsorption technology, while it can be used to “treat waste with waste,” the technology for regenerating saturated adsorbents is not yet mature, and there remains a potential risk of pollutant release during final disposal [16,96,99]. An innovative approach is to directly convert bottom ash into adsorbent materials like tobermorite via hydrothermal treatment, which creatively combines pretreatment with end-of-pipe management and opens a new avenue for resource recovery. Consequently, in practical applications, the choice of a pretreatment technology should not be made in isolation. It requires a forward-looking assessment of its compatibility and synergistic potential with downstream processes. By constructing an efficient, low-environmental-impact technology chain that integrates “pretreatment—stabilization/resource recovery,” we can address the challenges of secondary pollution and achieve a closed-loop process from a systems-level perspective.

In summary, to ensure efficient synergy between pretreatment and subsequent risk control, it is essential to systematically evaluate the characteristics and integration potential of the various technologies. For a clear and practical comparison, Table 6 summarizes the key parameters, environmental impacts, and potential integration pathways for each pretreatment technology with downstream processes.

## 5. An Integrated Study on Pathways for Risk Control and Valorization in MSWIBA Management

In contemporary practice, the management of MSWIBA has given rise to two divergent technical philosophies: a “risk-first” approach and a “resource-value-first” approach. This fundamental schism stems from differing priorities in the conceptualization of bottom ash’s essential characteristics, leading to the development of entirely distinct technical paradigms and evaluation metrics [40]. The “risk-first” strategy places environmental safety at the forefront, favoring techniques such as solidification/stabilization and thermal treatment to prioritize the conversion of bottom ash into an inert, environmentally benign material before considering its lower-grade applications [14,35,87]. While this approach provides a high degree of risk certainty and aligns with stringent regulatory requirements, it is often associated with prohibitively high processing costs and may, through “over-stabilization,” permanently forfeit the material’s latent potential, including its cementitious activity and valuable metal content [65]. Conversely, the “resource-value-first” approach emphasizes material circularity and economic viability. It advocates for the primary recovery of metal resources through methods like physical separation and hydrometallurgy [41,45,48], or for the direct utilization of ash’s physicochemical properties in building material production, integrating risk management into the utilization process itself [75,76]. Although this path enhances resource efficiency, it can harbor a legacy of “deferred risk” due to an insufficient understanding of long-term leaching behaviors and the safety of products over their service life, potentially resulting in short-term economic gains that do not offset future environmental liabilities [4].

**Figure 1:**
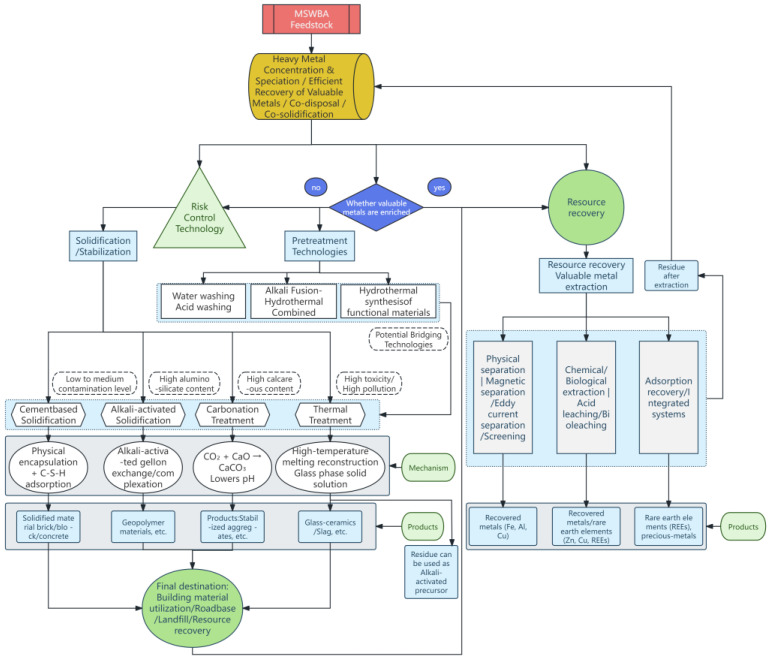
MSWIBA Treatment and Resource Utilization Technology Pathway and Decision Flowchart.

The prolonged coexistence and opposition of these two strategies highlight the inadequacies of traditional linear thinking when addressing such complex “resource-risk hybrids.” Neither a “first, make it safe, then use it” nor a “first, use it, then manage it” strategy successfully reconciles safety and efficiency from a systemic perspective. Indeed, characterizing MSWIBA with a single attribute—as either purely waste or purely resource—fails to capture its highly heterogeneous nature. Consequently, future advancements necessitate a move beyond this binary choice toward a dynamic, multi-path, and collaborative systems-integration framework founded on a granular understanding of material properties. The core of this new logic is to shift from seeking a single, universal “optimal” treatment process to constructing a flexible “divert-collaborate-optimize” framework (as illustrated in Figure 1).

The decision-making framework for MSWIBA treatment and resource recovery, presented in Figure 1, is a tangible embodiment of this integrated logic. The framework initiates with a multi-dimensional assessment of the bottom ash’s characteristics. By concurrently diagnosing its heavy metal speciation, valuable metal enrichment, chloride content, particle size distribution, and pozzolanic reactivity, it enables intelligent material classification. Based on the principle of “controllable risk, maximized value,” the framework then directs the material into multiple pathways: streams enriched with valuable metals (notably precious and rare earth metals) are channeled into a deep resource recovery circuit; streams with a suitable chemical composition and strong cementitious properties, following appropriate pre-treatment, enter a building material utilization circuit; and streams presenting significant pollution risks are directed into a deep stabilization circuit. Crucially, Figure 1 demonstrates that these circuits are not siloed but are interconnected through synergistic material and energy loops. For instance, residues from thermal treatment can serve as precursors for alkali-activated materials, and post-metal-recovery tailings, after stabilization, can be utilized as fillers. This internal resource circulation enhances the overall efficiency of the system.

The implementation of this integrated framework signifies a profound transition in the management paradigm, shifting from “static path selection” to “dynamic system construction.” It dictates that future technological advancements must not only concentrate on breakthroughs in individual processes but must also prioritize the enhancement of system integration capabilities. This encompasses the development of modular, intelligent equipment to accommodate the dynamic variations in material flow, the establishment of an end-to-end data monitoring and decision support platform to enable precise scheduling, and the application of life cycle assessment tools to perpetually optimize the system’s overall sustainability. Only through this comprehensive system reconfiguration can the objectives of resource valorization and risk control be effectively harmonized, thereby advancing the management of MSWIBA toward a future that is safe, efficient, and sustainable.

## 6. Conclusions

This study provides a systematic review of the physicochemical properties, value-added utilization pathways, and risk management strategies for MSWIBA. The findings reveal that MSWIBA is characterized by a complex and heterogeneous composition, containing significant amounts of silicon, calcium, and aluminum oxides, along with various heavy metals. Its leaching behavior is notably influenced by pH levels, particle size distribution, and mineralogical phase. Regarding resource utilization, MSWIBA can serve as a supplementary cementitious material or aggregate substitute in cement-based composites, road construction, and masonry products. Concurrently, valuable metals—including iron, aluminum, copper, and rare earth elements—can be effectively recovered through methods such as physical separation, hydrometallurgy, and bioleaching. For environmental risk mitigation, techniques like solidification/stabilization, carbonation, and adsorption have proven effective in controlling the migration and release of heavy metals.

Nevertheless, the field confronts several persistent challenges: the natural heterogeneity of MSWIBA results in inconsistent product quality; the long-term environmental behavior of heavy metals and the durability of stabilized forms remain inadequately understood; current technologies are often siloed, lacking integrated, end-to-end system approaches; and the economic viability and scalability of certain technologies require further validation.

In conclusion, future research on MSWIBA should prioritize the transition from fragmented technologies to integrated systems. The critical first step is to develop advanced characterization and classification methodologies based on rapid sensing and intelligent recognition to inform optimal resource utilization pathways. Building on this foundation, it is imperative to establish a synergistic “recovery-stabilization-utilization” framework, which hinges on transcending conventional process boundaries to foster cross-technological innovation. A prime example involves employing vitrified residues from thermal treatment as highly reactive precursors for alkali-activated cements, thereby creating a closed-loop system that concurrently achieves stabilization and high-value outcomes, thus augmenting product worth while also safeguarding environmental integrity. Furthermore, it is essential to deepen fundamental understanding of the material’s long-term environmental behavior, develop eco-efficient and durable stabilization materials and processes, and construct a comprehensive life cycle assessment framework incorporating environmental, economic, and carbon metrics. These measures will provide the scientific foundation necessary to transform MSWIBA’s paradigm from “urban waste” to “sustainable resource,” ultimately enabling the circular and low-carbon advancement of solid waste management.

## Figures and Tables

**Table 1:** Chemical composition of bottom ash [17].

Element Name	XRF	SEM-EDS
Al	4.7	3.97
C	-	9.96
Ca	33.4	22.91
Cl	2.9	2.5
Cr	0.1	-
Cu	0.2	-
Fe	5.7	5.09
K	1.5	0.52
Mg	0.9	0.46
Mn	0.2	-
Na	-	0.62
O	36.1	47.25
P	1.3	-
S	2.6	3.62
Si	5.8	1.34
Sr	0.1	-
Ti	3.8	1.77
Zn	0.6	-
Zr	0.1	-

Note: 1. The data presented above are expressed as mass percentages. 2. A hyphen (“-”) denotes that the substance was not detected.

**Table 2:** Typical Ranges of Principal Oxide Compositions in MSWIBA from Different Regions.

Country/Region	SiO_2_	CaO	Al_2_O_3_	Fe_2_O_3_	Data Source
China	28.9–41.52	14.9–33.5	1.7–8.4	4.1–8.64	[25,27]
China (Shenzhen)	37.6	28.7	7.35	6.32	[28]
Japan	34.7–39.9	11.1–18.2	12.3–16.5	7.1–8.6	[25]
United States	39.2–44.7	10.5–14.8	17.0–17.4	9.2–10.4	[25]
Italy	52	8.1	28	5	[25]
Netherlands (BAW Plant)	54.2	13.4	7.9	13.8	[26]
Netherlands (BAM Plant)	51.4	13.9	7.9	15.1	[26]
India	54.43	18.71	10.11	5.29	[29]

Note: 1. The data presented above are expressed as mass percentages. 2. The data presented are derived from specific studies and reflect the measured compositions of samples collected at the indicated locations. They illustrate typical characteristics under local waste management and incineration conditions, rather than strict national averages. Variability within regions may exist due to differences in waste composition, seasonal factors, and treatment technologies.

**Table 3:** Comparison of Key Recovery Technology Features for Valuable Metals in MSWIBA.

Technology Type	Applicable Metals	Core Advantages	Major Disadvantages	Representative Studies/Processes
Physical separation	Fe, Al, Cu (enriched in >2 mm coarse particles)	Simple process, low operating cost, high separation efficiency	Poor recovery of fine metal particles, relies on particle size pre-classification	[41]
Chemical leaching	Zn, Cu, Pb, Rare Earth Elements (REEs, suitable for fine particles)	High extraction rate (can exceed 80%), suitable for fine-grained materials	Prone to generating heavy metal-containing wastewater, requires subsequent treatment processes	[45]
Electrochemical extraction	Multiple metals (Zn, Cu, Cd, etc.)	Strong selectivity, high purity of recovered products (can exceed 90%)	Complex equipment structure, high operating energy consumption	[1]
Bioleaching	Zn, Cu, Cd, etc.	Environmentally friendly, low energy consumption, adaptable to complex systems	Long reaction cycle, efficiency highly dependent on microbial activity and environmental conditions	[48]
Adsorption recovery (e.g., MMS-PP)	Rare Earth Elements (REEs), Precious metals	Excellent selectivity, adsorbent is recyclable	High adsorbent preparation cost, complex regeneration process	[47]
Integrated system (e.g., three-step process)	Rare Earth Elements (REEs), Precious metals	Integrates resource recovery with waste detoxification, low environmental impact	Complex process chain, demanding operation and control requirements	[13]

**Table 4:** Comparative Analysis of MSWIBA Utilization in Construction Materials.

Application Field	Concrete Utilization Method	Typical MSWIBA Dosage/Treatment	Main Advantages/Mechanism of Action	Limitations and Challenges Reference	Data Source
Cementitious Materials	As a cement replacement	15% (OPC replacement); 30% (cement replacement); 10–25% (SBA/VA replacement)	Possesses pozzolanic activity, participates in hydration; reduces carbon emissions; performance can be optimized by compounding with GGBS, RHA, etc.	Significant strength reduction at high dosage; hydration retardation; requires additive assistance; potential heavy metal leaching risk	[5,6,9,56,57,58,59]
	As aggregate and filler	0–100%(by volume) replacement of natural aggregate	Improves microstructure; porous structure buffers freeze–thaw stress; highly active cements like CEM III enhance interfacial bonding	Irregular particle shape, rough surface, high water absorption; high dosage leads to degradation of mechanical and durability properties; ASR risk	[5,60,61,62,63,64,65,66,67,68]
	Alkali-activated cementitious materials (Geopolymer)	Alkali activation system (e.g., NaOH, water glass)	Silico-aluminous reactivity forms cementitious phases under strong alkali; heavy metals are solidified in the gel structure; enables low-energy curing	Reaction heavily influenced by pH, Si/Al ratio, calcium source; high alkalinity may inhibit hydration; stringent process control required	[69,70,71,72,73,74]
Road Engineering Materials	Asphalt mixture (filler substitute)	Substitute limestone filler, dosage gradually increased to 100%	Porous structure adsorbs asphalt; surface chemistry may promote interfacial bonding; weak pozzolanic reaction may provide long-term structural stability	Smooth, spherical particles lead to weak interfacial bonding; significant fatigue performance decline; requires composite use with RAS, etc., to compensate for asphalt	[8,75,76,77]
	Cement-stabilized base	30–45% aggregate replacement	CaO, SiO_2_ participate in cement hydration, forming more C-S-H; porous structure buffers freeze–thaw stress	Dosage must be controlled to avoid strength loss; long-term durability requires further verification	[7]
	Permeable subgrade material	As aggregate to form interconnected pore structure	Particle gradation forms connected pores; porous surface provides physical and chemical adsorption sites	Generally lower strength, suitable for non-load-bearing permeable structures; long-term clogging risk needs consideration	[34]
Bricks, Tiles & Other Products	Sintered bricks (Traditional sintering)	5–30% clay replacement, sintering at 900–1000 °C	Ca, Fe, etc., form low-temperature eutectic phases aiding sintering; increased porosity can reduce thermal conductivity	High dosage increases pore connectivity, reducing durability and frost resistance	[78,79]
	Alkali-activated bricks (Chemical activation)	NaOH pretreatment + Ca(OH)_2_ activation, cured at ambient temperature	Pre-removal of Al prevents expansion; Ca(OH)_2_ provides calcium source, promoting formation of dense C-(A)-S-H gel; reduces energy consumption by 30.6–44.2%	Performance sensitive to activator type and calcium source; requires control of alkalinity and Si/Al ratio	[27]
	Composite/Modified bricks	Composite with cement, plastics, fly ash, etc.	Cement hydration provides C-S-H; plastics form a continuous coating network; fly ash optimizes gradation for self-compaction	Complex mix design; plastic bonding requires control of melting temperature and dispersion uniformity	[29,80,81]
	Glass-ceramic tiles (High-temperature phase transformation)	Vitrification treatment (>1000 °C), controlled crystallization	High-temperature melting reconstructs phases, forming a dense structure of glass matrix reinforced by crystalline phases	High energy consumption; strict process control; higher cost	[82]

Note: OPC = Ordinary Portland Cement; SBA = Sintered Bottom Ash; VA = Vitrified Ash.

**Table 5:** Performance and Suitability of Various Solidification Techniques for Heavy Metal Stabilization in MSWIBA.

Technology Type	Core Mechanism	Main Advantages	Main Disadvantages	Applicable Conditions	Typical Application Scenarios	Data Source
Cement-based solidification	Physical encapsulation, adsorption, and precipitation within hydration products (e.g., C-S-H gel)	Low cost, mature process, synergistic use in construction materials, solidified body possesses certain structural strength	Chlorides/sulfates interfere with hydration, increased volume of solidified body, long-term stability requires further verification	Suitable for treating bottom ash with low to medium contamination levels	Construction products (bricks, blocks), roadbed filler materials, landfill cover layers	[69,84,85]
Alkali-activated solidification	Silico-aluminous components form gel network under alkali action, fixing heavy metals via ion exchange and surface complexation	High solidification efficiency (>98% fixation rate), excellent durability, capable of co-processing multiple ash types	Higher cost of alkali activators, strict process parameter control, long-term environmental impact requires ongoing monitoring	Suitable for bottom ash with high silico-aluminous content	Co-disposal of hazardous waste, geopolymer building material production, applications demanding high performance of solidified bodies	[69,71]
Thermal treatment (Sintering/Vitrification)	High-temperature reconstruction of mineral phases, dissolving heavy metals into glassy phases or stable crystalline structures	Excellent heavy metal fixation, significant volume reduction in solidified body, achieves complete detoxification	Energy-intensive, high capital and operating costs, limited scalability	Suitable for treating highly toxic or heavily contaminated bottom ash	Centralized disposal of hazardous waste, production of speciality construction materials (glass ceramics), scenarios with extremely high detoxification requirements	[86,88,89]
Carbonation treatment	CO_2_ reacts with calcareous components to form carbonates, lowering system pH and promoting heavy metal precipitation/adsorption	Relatively low cost, enables CO_2_ utilization (carbonation), carbon reduction potential, simple process operation	Limited effectiveness for anionic heavy metals (e.g., Sb, Cr), long-term stability requires field validation	Suitable for bottom ash with high calcareous (CaO) content	Pre-treatment of construction aggregates, carbon-neutral technology pathways, stabilization pre-treatment of low-risk bottom ash	[14,35,36]

**Table 6:** Table of Pretreatment Technologies and Their Compatibility with Subsequent Disposal.

Pretreatment Technology	Core Treatment Objective	Key Parameters and Treatment Effectiveness	Environmental Impact and Control	Potential Downstream Integration Technology	Application Priority	Data Source
Water Washing Pretreatment	Removal of soluble chlorides and partially soluble heavy metals, eliminating interference for solidification	Liquid-to-solid ratio 3:1, stirring at room temperature for 30 min, chloride removal rate 68.4–75.0%, heavy metal removal rate 10.28–19.38%	Generates high-salinity wastewater; requires salt recovery via evaporation/crystallization; no secondary pollution	Serves as a preliminary step, often combined with cement-based solidification or carbonation treatment	High (simple process, wide applicability, preferred choice)	[90,93]
Acid Washing Pretreatment	Deep removal of heavy metals (e.g., Zn, Cu, Ni), reducing subsequent disposal load	HCl concentration 5–10%, liquid-to-solid ratio 4:1, Zn/Cu/Ni removal rate 62–76%, requires pH adjustment/stabilization with MgO	Highly corrosive; requires corrosion-resistant equipment; acidic wastewater must be neutralized before discharge	Potentially beneficial for downstream thermal treatment (sintering/vitrification) or high-demand alkali-activated solidification	Medium (targeted, suitable for high-risk scenarios)	[95]
Alkali Fusion–Hydrothermal Combined Treatment	Simultaneous chloride removal and valorization, converting bottom ash into functional material precursors	Alkali fusion temperature 600–700 °C, hydrothermal temperature 180–200 °C, chloride removal rate near 100%, capable of synthesizing zeolites	Relatively high energy consumption; no wastewater accumulation; alkali fusion slag can be fully utilized	Its products can be used for alkali-activated solidification or as raw materials for hydrothermal synthesis of functional materials	Medium-Low (for high-value scenarios, scalability limited)	[15]
Hydrothermal Synthesis of Functional Materials	Upgrading bottom ash into value-added heavy metal adsorbents	Hydrothermal temperature 180 °C, reaction time 12 h, synthesizes tobermorite, Cu(II) adsorption capacity 270.3 mg/g	Controllable reaction conditions; products are recyclable; generates no secondary waste	Its products can be directly used for heavy metal adsorption in water treatment, achieving bottom ash resource recovery	Low (for specialized functional needs, non-conventional risk control)	[96]

## Data Availability

No new data were created or analyzed in this study. Data sharing is not applicable to this article.
